# The Rising Burden of Diabetes and Hypertension in Southeast Asian and African Regions: Need for Effective Strategies for Prevention and Control in Primary Health Care Settings

**DOI:** 10.1155/2013/409083

**Published:** 2013-03-14

**Authors:** Viswanathan Mohan, Yackoob K. Seedat, Rajendra Pradeepa

**Affiliations:** ^1^Madras Diabetes Research Foundation and Dr. Mohan's Diabetes Specialities Centre, WHO Collaborating Centre for Noncommunicable Diseases Prevention and Control, IDF Centre for Education, 4 Conran Smith Road, Gopalapuram, Chennai 600 086, India; ^2^Nelson R Mandela School of Medicine, University of KwaZulu-Natal, 4013 Congella, Durban, South Africa

## Abstract

*Aim*. To review the available literature on burden of diabetes mellitus (DM) and hypertension (HTN) and its coexistence in Southeast Asian (SEA) and the African (AFR) regions and to suggest strategies to improve DM and HTN prevention and control in primary health care (PHC) in the two regions. *Methods*. A systematic review of the papers published on DM, HTN, and prevention/control of chronic diseases in SEA and AFR regions between 1980 and December 2012 was included. *Results*. In the year 2011, SEA region had the second largest number of people with DM (71.4 million), while the AFR region had the smallest number (14.7 million). Screening studies identified high proportions (>50%) of individuals with previously undiagnosed HTN and DM in both of the SEA and AFR regions. Studies from both regions have shown that DM and HTN coexist in type 2 DM ranging from 20.6% in India to 78.4% in Thailand in the SEA region and ranging from 9.7% in Nigeria to 70.4% in Morocco in the AFR region. There is evidence that by lifestyle modification both DM and HTN can be prevented. *Conclusion*. To meet the twin challenge of DM and HTN in developing countries, PHCs will have to be strengthened with a concerted and multipronged effort to provide promotive, preventive, curative, and rehabilitative services.

## 1. Introduction

 Diabetes mellitus (DM) and hypertension (HTN) have emerged as major medical and public health issues worldwide, and both are important risk factors for coronary artery disease (CAD), heart failure, and cerebrovascular disease. DM is increasing in epidemic proportions globally. According to the WHO, the prevalence of DM in adults worldwide was estimated to be 4.0% in 1995 and is predicted to rise to 5.4% by the year 2025 such that the number of adults with DM in the world would rise from 135 million in 1995 to 300 million in the year 2025 [1]. The recent Burden of Metabolic Risk Factors of Chronic Diseases Study [2] conducted in 199 countries worldwide to assess the national, regional, and global trends in diabetes reported that the age-standardized adult diabetes prevalence was 9*·*8% (8.6–11.2) in men and 9.2% (8.0–10.5) in women in 2008, up from 8.3% (6.5–10.4) and 7.5% (5.8–9.6) in 1980. The number of people with diabetes increased from 153 (127–182) million in 1980 to 347 (314–382) million in 2008. The International Diabetes Federation (IDF) has come up with much higher figures in a recent report which estimated that in 2011, 366 million people worldwide had DM and if this trend continues, by 2030, 552 million people, or one in 10 adults, will have DM [3]. In the same report, the Western Pacific Region was shown to have the largest number of people with DM (132 million), followed by Southeast Asian (SEA) region (71.4 million), while the African (AFR) region had the smallest number (14.7 million) [3]. DM exerts a significant burden resulting in increased morbidity and mortality, decreased life expectancy, and reduced quality of life, as well as individual and national income losses.

Additionally, HTN affects about one billion people worldwide [4] and it is estimated that by 2025, up to 1.56 billion adults worldwide will be hypertensive [5]. Raised blood pressure (BP) is estimated to cause 7.5 million deaths, which accounts for 57 million disability-adjusted life years (DALYs). The Burden of Metabolic Risk Factors of Chronic Diseases Study reported that in 2008, age-standardised mean SBP worldwide was 128.1 mm Hg (95% uncertainty interval 126.7–129.4) in men and 124.4 mm Hg (123.0–125.9) in women. Globally, between 1980 and 2008, SBP decreased by 0.8 mmHg per decade in men and 1.0 mmHg per decade in women [6]. The high prevalence of HTN makes it a significant factor for mortality and morbidity. Individuals with HTN are known to have a twofold higher risk of developing CAD, four times higher risk of congestive heart failure, and seven times higher risk of cerebrovascular disease and stroke compared to normotensive subjects. According to global estimates 62% of stroke, 49% of CAD, and 14% of other nonfatal cardiovascular disease (CVD) events are attributed to nonoptimal BP. It has been reported that the highest number of deaths and DALYs attributable to high BP occurred in the WHO Western Pacific (WP) and SEA regions [7]. Extensive epidemiological studies conducted in AFR region show that HTN is one of the commonest cardiovascular ailments and that BP assumes more importance with increasing age, particularly in the Sub-Saharan Africa [8].

DM and HTN are also known to coexist in patients [9]. Indeed, there is a strong correlation between changing lifestyle factors and increase in both DM and HTN. Challenges in managing both DM and HTN more effectively include factors at the patient, provider, and system levels. Epidemiologic studies have an important clinical impact and have led to an increasing appreciation of the value of epidemiology as a scientific basis for clinical and public health practice. As primary health care is the first level of contact of the individuals, the family, and the community with the national health system, there is an urgent need for an integrated approach at primary health care (PHC) level for addressing the burden of HTN and DM. The aim of this paper is to review the available literature on the burden of DM and HTN in the SEA and AFR regions and to suggest strategies to improve DM and HTN prevention and control in primary care in the two regions.

## 2. Burden of Diabetes in SEA and AFR Regions

According to the World Health Organization (WHO), DM, a potentially life threatening disorder, is considered as “an apparent epidemic which is strongly related to life style and economic change” [10]. Type 2 DM comprises 90–95% of all cases of DM, and it increases the risk of both macrovascular diseases (which comprise CAD and cerebrovascular disease or “stroke,” and peripheral vascular disease) and microvascular diseases (retinopathy, nephropathy, and neuropathy). Diabetes creates a huge economic burden not only due to the direct costs of treatment particularly of its complications, but also in terms of man hours lost due to the debilitating effect the disease has on the individual and his or her family and society as a whole.

**Figure 1:**
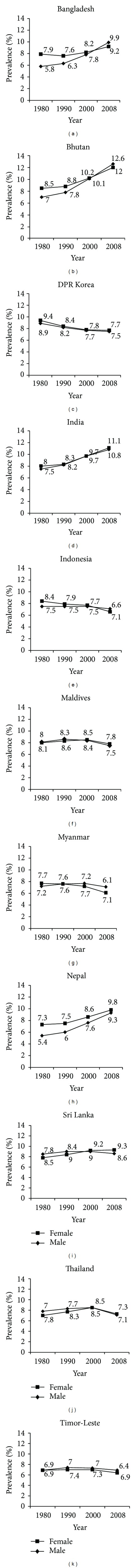
Trends in age-standardised diabetes prevalence in the SEA region between 1980 and 2008 for male and female population [2].

Epidemiological studies have shown that DM is increasing rapidly in people of South Asian, African, and African Caribbean origins [11]. The WHO and IDF have utilized methods that combine available country data at regular intervals and extrapolated estimates for remaining countries without data. In 2000, according to WHO, the prevalence of DM among the 46 countries of the WHO AFR region was estimated at 7.02 million people and among the 11 countries of the SEA region to be 4.69 million [12]. The number of individuals with DM in the SEA region in the year 2011 and the projected figures for the year 2030 are presented in Table 1 [3]. There are significant differences between and within countries because of the geographical diversity in socioeconomic growth rates, demographic and lifestyle changes, and perhaps differences in ethnic susceptibility to DM. For example, the prevalence of DM in Sub-Saharan Africa ranges from 1% in rural Uganda to 12% in urban Kenya [13, 14] and 8.1% in urban and 2.3% in rural Bangladesh [15]. In India, the recent ICMR-INdia DIABetes (ICMR-INDIAB) Study reported the prevalence of DM (both known and newly diagnosed) in 4 regions of the country: 10.4% in Tamilnadu, 8.4% in Maharashtra, 5.3% in Jharkhand, and 13.6% in Chandigarh (Union Territory) [16]. The overall number of people with DM in India in 2011 based on this study was estimated to be 62.4 million [16], and this was confirmed by the Diabetes Atlas (5th edition) of the IDF, which gave a figure of 61.3 million people with DM in India in the age group of 20–79 years [3].  Figure 1 presents the trends in age-standardised DM prevalence in the SEA region between 1980 and 2008 for men and women as reported in the Burden of Metabolic Risk Factors of Chronic Diseases Study [2].

The recent NFHS 3 data [17], which studied urban and rural residents (all women aged 15–49 and all men aged 15–54) in 29 states of India during the year 2005-2006, reported that more than two percent of men and women had self-reported DM. The number of women who had DM ranged from 282 per 100,000 women in Rajasthan to 2,549 per 100,000 women in Kerala. In five other states (Tamil Nadu, Goa, Tripura, West Bengal, and Delhi) the number of women with DM was relatively high (above 1,500 per 100,000 women). Only five states had DM prevalence levels below 500 per 100,000 men (Jammu and Kashmir, Mizoram, Himachal Pradesh, Rajasthan, and Uttar Pradesh).

It has also been reported that there is a dramatic increase in the prevalence of type 2 DM in the African regions [18]. In studies done between the 1960s and early 1980s, prevalence of type 2 DM was mostly lower than 1%, except in South Africa and Côte d'Ivoire [19]. However, currently DM rates are rising in many parts of Africa [20]. According to the Burden of Metabolic Risk Factors of Chronic Diseases Study, the DM prevalence in 2008 was higher in North Africa and was the lowest in Sub-Saharan Africa [3] and the prevalence ranged from 1% in rural Uganda to 12% in urban Kenya [21]. Studies from Sub-Saharan Africa report prevalence rates of 2.5–8% in rural and urban communities in West Africa [22–24] and 1–12% in rural and urban communities in East Africa [14, 25–27]. Prevalence rates of 3–10% were noted in urban and rural populations in South Africa [28, 29], while in Central Africa the figures ranged from 2.9% to 6.2% in rural and urban communities [30, 31] which are comparable with rates in developed countries. In a recent community-based screening for DM conducted simultaneously in four major Cameroonian cities, the sex-specific-age-adjusted prevalence of DM (for men and women) had dramatically risen to 10.1% and 11.2%, respectively [32]. The number of individuals with DM in the AFR region in the year 2011 and the projected figures for the year 2030 are presented in Table 2 [3].

One of the unfortunate aspects of DM is that more than half of the people with DM are unaware of the condition. The rate of undiagnosed DM is high in both of the SEA and AFR regions. In the ICMR-INDIAB Study the prevalence of undiagnosed DM among residents of Tamil Nadu, Maharashtra, Jharkhand, and Chandigarh were 44%, 70%, 51%, and 50% [16]. The AFR region has the highest proportion of undiagnosed DM, at least 78% [3].

## 3. Burden of HTN in SEA and AFR Regions

In developed countries of the world, the prevalence of HTN is beginning to stabilize or decrease, thanks to prevention and control measures [33]. Parallelly the prevalence rates of CVD (both CAD and stroke) are also beginning to fall [34, 35]. In contrast, in the developing regions of the world such as SEA and AFR hypertension and CVD rates continue to rise. Extensive epidemiological studies show that HTN is one of the commonest cardiovascular ailments in AFR and SEA regions. Approximately one-third of the adult population has high BP and nearly 1.5 million deaths occur due to HTN each year in the SEA region. Studies in India have shown an increasing trend in the prevalence of HTN among urban adults: men 30%, women 33% in Jaipur (1995); men 44%, women 45% in Mumbai (1999); men 31%, women 36% in Thiruvananthapuram (2000); men 36%, women 37% in Jaipur (2002); 21.1% in Chennai (2003). Among the rural populations, HTN prevalence in men was 24% and in women 17% in Rajasthan (1994) [36]. The CURES Study reported a prevalence of 23.2% HTN among men and 17.1% among women in South India [37]. HTN is responsible for 57% of all stroke mortality and 24% of all CAD mortality in India. In Thailand, the age-adjusted prevalence of HTN was 25% in men and 24% in women [38]. In Bangladesh, prevalence rates of systolic and diastolic HTN were 14.4 and 9.1 percent, respectively, with prevalence of systolic HTN significantly higher in rural than in urban participants [39].

In addition, there is an approximately 89% increase in Sub-Saharan Africa from 2000 through 2025 versus a 24% increase in more developed countries. Among the WHO regions, the prevalence of HTN was the highest in Africa, where it was 46% for both sexes combined. Both men and women have high rates of raised BP in the African region, with prevalence rates over 40% [40]. It has been reported that the prevalence of HTN is increasing rapidly in Sub-Saharan Africa and occurring in young and active adults [8]. Age-adjusted prevalence urban studies have shown that in the adult population of Durban, HTN was the highest in Zulus (25%), intermediate in Whites (17.2%), and the lowest in Indians (14.2%). However, in contrast, the prevalence of HTN in rural areas was lower: 4.1% in Ghana, 5.9% in Nigeria, 7% in Lesotho, and 7.37% in the rural Zulu [41]. 

A recent systematic analysis of health examination surveys and epidemiological studies in 199 countries and territories with 786 country-years and 5.4 million participants has looked at the national, regional, and global trends in systolic BP (SBP) for adults 25 years and older since 1980 [6]. The SBP rose in Oceania, East Africa, and South and Southeast Asia for both sexes and in West Africa for women, with the increases ranging from 0.8 to 1.6 mmHg per decade in men and 1.0 to 2.7 mmHg per decade in women.  Table 3 shows the trends in age-standardised mean SBP in selected African region between 1980 and 2008 for male and female population as reported in the Burden of Metabolic Risk Factors of Chronic Diseases Study [6].

Control of HTN in Sub-Saharan Africa is poor and target BP <140/90 mmHg on treatment occurred in <1% in Tanzania [42] and 7% in Black males and 15% in Black females in South Africa [43]. Obstacles to adherence include (a) long duration of therapy, (b) complicated regimens, (c) cost of drugs, (d) side effects of medication, (e) lack of specific appointment time, (f) long waiting period at clinic or office, (g) lack of consistent and continuous primary care, (g) instructions not understood, (h) organic brain damage (memory deficit), and (i) medicines not available. The patient and illness characteristics include (a) asymptomatic nature of the condition; (b) chronic conditions require constant attention; (c) there are no immediate consequences of stopping treatment for example, one does not feel sick; (d) social isolation; (f) disrupted home situation; and (g) psychiatric illnesses.

Significant numbers of individuals with HTN in both of the SEA and AFR regions are unaware of their condition and, among those diagnosed with HTN, treatment is frequently inadequate. HTN was estimated to have caused 46,888 deaths or 9% of all deaths and 390,860 DALYs or 2.4% of all DALYs in South Africa in 2000. Overall, 50% of stroke, 42% of IHD, 72% of hypertensive disease, and 22% of other CVD burdens in adult males and females (30+ years) were attributable to high BP [44]. In Thailand, burden of diseases defined as total DALY loss attributed to HTN was 5.5% in both men and women [45].

## 4. Coexistence of Diabetes and Hypertension

HTN is a common comorbid condition in DM and vice versa. DM and HTN coexist in approximately 40% to 60% of patients with type 2 DM [46, 47]. HTN may precede the onset of DM. In about 95% cases, it is essential HTN and the rest may be secondary HTN. In some cases, both HTN and DM may be present at the time of initial diagnosis [48]. The Hypertension in Diabetes Study (HDS)-I conducted to determine the prevalence of HTN in newly diagnosed type 2 diabetic patients reported that 39% of the patients were hypertensive at the time of diagnosis of DM [49].

There is considerable evidence for an increased prevalence of HTN in diabetic persons from other populations [50]. In a study conducted in American Indian and Alaska Native communities to estimate the prevalence of clinical HTN and assess the coexistence with DM, 37% of diabetic individuals were diagnosed with HTN [51]. In the same study, the relative risk of HTN in the diabetic populations compared with the nondiabetic populations varied from 4.7 to 7.7 [52]. It has also been shown that in hypertensive patients agaging 40–59 years, of three different ethnic groups living in South London, are more likely to have DM, and, conversely, patients with DM have a greater chance of having HTN [52]. In a large prospective cohort study that included 12,550 adults, the development of type 2 DM was almost 2.5 times as likely in persons with HTN than in their normotensive counterparts [50, 53]. 

Both essential HTN and DM affect the same major target organs. The common denominator of hypertensive/diabetic target organ-disease is the vascular tree. People with coexisting DM and HTN are at increased risk of developing atherosclerosis, retinopathy, renal failure, and nontraumatic amputations and CVD [54]. Moreover, it has been shown that lowering BP in high risk patients with DM can reduce deaths from strokes, overall mortality, and CVD events and can slow the progression of renal disease in patients with type 2 DM [55]. Left ventricular hypertrophy and CAD are much more common in diabetic hypertensive patients than in patients suffering from HTN or DM alone [56]. HTN substantially increases the risk of both macrovascular and microvascular complications in DM. In studies such as the Systolic Hypertension in the Elderly Program (SHEP) and the Systolic Hypertension in Europe Study (Syst-Eur), those with coexisting DM had an approximate doubling in cardiovascular morbidity and mortality [55, 57].

Studies show that glycaemic control is effective in reducing microvascular complications. However “tight” HTN control appears to be more effective than glycaemic control in reducing microvascular events particularly kidney disease. Clinical trials have demonstrated the importance of intensive treatment of HTN among patients with DM. The UKPDS showed that each 10 mmHg decrease in mean SBP was associated with 12% reduction in the risk for any complication related to DM, 15% reduction in deaths related to DM, 11% reduction in myocardial infarction, and 13% reduction in microvascular complications [58].

The Hypertension in Diabetes Study (HDS)-II [59], which looked at the degree to which HTN is a risk factor for macrovascular and microvascular complications in type 2 DM, reported that hypertensive patients had a greater incidence than normotensive patients of death from diabetes-related, mainly cardiovascular events and a greater incidence of diabetes-related death and major morbidity combined, including myocardial infarctions, angina, strokes, and amputation. 

Studies from SEA region have shown that DM and HTN coexist in type 2 DM. In India about 50% of diabetic individuals have HTN [60, 61]. In the Screening India's Twin Epidemic (SITE) cross-sectional study conducted in 10 Indian states [62] DM and HTN were coexistent in 20.6% patients, which demonstrate that the substantial burden of DM and HTN is on the rise in India. According to the Thailand Diabetes registry, the prevalence of HTN in adult Thai type 2 diabetic patients was 78.4 [63]. Among 745 subjects with known DM, who participated in the Fourth Korea National Health and Nutrition Examination Survey in 2007 and 2008, the prevalence of HTN was reported to be 55.5% [64]. In a study conducted in seven urban populations of Nepal, 36.7% of the diabetic subjects had HTN, while of all subjects with HTN, 29.1% had DM (known or newly diagnosed) [65].

African studies have reported high prevalence of HTN in DM, although most are clinic based. A high prevalence rate of 66.4% was reported in Cameroonian type 2 diabetic population (African Blacks) [66]. A study conducted in Kenya during the year 2005 to determine the proportion of specific cardiovascular risk factors in ambulatory patients with type 2 DM reported that 50% of the patients had HTN [67]. In a Nigerian clinic population, HTN and DM coexisted in 9.7% of the patients studied [68]. The recent EPIDIAM Study conducted in 525 type 2 diabetic individuals in three Moroccan regions reported that 70.4% of the individuals had HTN [69]. Another study conducted in Benin City, Nigeria, reported that 54.2% of the diabetic individuals had HTN [70]. 

## 5. Strategies and Interventions to Prevent/Control Diabetes and Hypertension in Primary Care Settings

In most developing countries, till recently, the priorities of health care had been the prevention and control of communicable diseases. However now the attention has begun to shift to the control and prevention of noncommunicable diseases (NCDs) including DM, HTN, CAD, and stroke in view of the rising trend of NCDs. For this, an integrated approach to the prevention and management, irrespective of cause, is needed in primary health care settings [71]. Chronic disease interventions selected for use in primary health care must lead to productive changes in risk status and outcomes, be cost effective, and be financially and logistically feasible, which are available for implementation across a range of resource settings [72].

**Figure 2:**
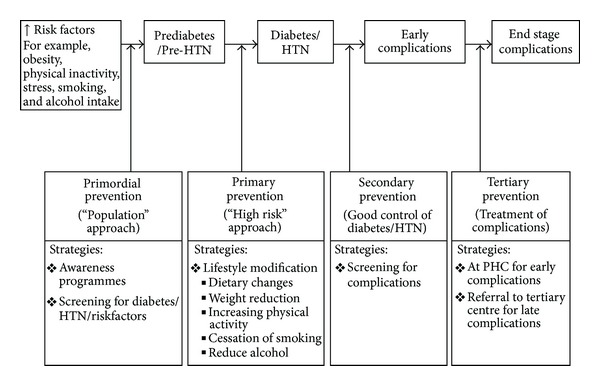
Strategies for prevention for diabetes and hypertension at different levels.

Within this context of restrained economic conditions, in the SEA and AFR regions, the greatest gains in controlling the DM and HTN epidemics lie in their prevention, or at least early detection and adequate control. For most low-income and middle-income countries, the major obstacle to the control of BP-related diseases is the absence of appropriate primary health care services [73]. The commonality of many risk factors for HTN and DM justifies an integrated approach to the prevention and control of both. Both problems have to be tackled at several levels, that is, primordial, primary, secondary, and tertiary prevention (Figure 2). “Primordial prevention” refers to reduction of the risk factors of DM/HTN and thereby decreasing the risk of developing DM in the future. “Primary prevention” refers to prevention (or postponement) of the condition in those in a prediabetes/HTN stage. “Secondary prevention” refers to prevention of complications in those who have already developed DM/HTN. Finally the term “Tertiary prevention” is used to describe limiting physical disability and preventing progression to end stage complications in those who have already developed some associated complications.

Primordial prevention depends on health policies that create a congenial environment and promote healthy behaviours and population wide education programs, which in turn depends on many factors, including political commitment, advocacy by health professionals, and involvement of community leaders and the mass media [74]. The “rule of halves” has shown that more than 50% of people with DM or HTN remain undiagnosed, among those detected, 50% do not take treatment, and finally among those who take treatment, over 50% are not under control. Thus overall only 6–8% of subjects with DM or HTN remain adequately controlled [75, 76]. This poses a huge challenge and underscores the need to urgently raise awareness in the community at large. Attempts should be made to detect DM/HTN early before irreversible organ damage occurs and to provide them with the best possible and yet affordable treatment. Beaglehole et al. [71] have emphasized that management of chronic diseases is fundamentally different from acute care, relying on several features including opportunistic case finding for assessment of risk factors, detection of early disease, identification of high risk status, a combination of pharmacological, and psychosocial interventions, often in a stepped-care fashion, and finally long-term followup with regular monitoring and promotion of adherence to treatment. Therefore, efforts should be made for detection through screening and early management at the community level. Screening activities are an important component of any prevention/control programme and are particularly important in population at high risk for developing DM/HTN (e.g., older individuals, some ethnic groups) and those with limited access to medical care (e.g., some minorities, migrants) (Figure 2). Luckily, unlike screening for some diseases, for example, cancer, screening for DM/HTN or prediabetes/HTN is fairly simple. In addition, a standardized algorithm of management can help in the rational use of available resources at the primary care level.

The next step after having the high risk group detected is to advise lifestyle modifications (primary prevention). While age and ethnicity are non-modifiable risk factors, physical inactivity and waist circumference, dietary and smoking habits, and so forth can be modified. Evidence from high-income settings shows that interventions to reduce risk behaviours (dietary modifications, increase exercise, and stop smoking) in people with serious risk factors or preexisting disease such as HTN, DM, and CVD are more successful in achieving reductions in risk factors and, in some circumstances, improving clinical outcomes [77]. The high risk group can be empowered through health education on lifestyle modification (LSM) and their benefits with the help of community health workers. 

Landmark studies like the Da Qing Study in China [78], Finnish Diabetes Prevention Study [79], and the Diabetes Prevention Programme (DPP) [80] have clearly shown that measures of LSM help in preventing DM. Both of the Finnish Diabetes Prevention Study and the DPP in the USA showed that lifestyle change can significantly reduce the risk of development of type 2 DM by 58% in individuals with IGT and demonstrated that modest weight change and achievable physical activity goals can translate into significant risk reduction [78, 79]. The Da Qing Study [78], conducted in a Chinese population, reported that diet, exercise, and diet-plus-exercise interventions were associated with 31%, 46%, and 42% reductions in risk of developing DM, respectively. The Indian Diabetes Prevention Programme (IDPP) showed that progression to DM from impaired glucose tolerance (IGT) can be prevented by 28.5% with LSM and 26.4% with with metformin (MET) in individuals who were younger, leaner, and more insulin resistant as compared with the control group [81]. However, there was no added benefit from combining LSM + MET (28.2%) [81].

There are also several clinical trials exploring the efficacy of lifestyle modifications to reduce BP. A prospective study on 8302 Finnish men and 9139 women aged 25 to 64 years without a history of antihypertensive drug use, CAD, stroke, and heart failure at baseline showed that subjects with heavy grade of physical activity had lower prevalence of HTN [82]. Of the three lifestyle changes—weight reduction, sodium reduction, and stress management tested in 2182 participants with prehypertension in the Trials of Hypertension Prevention-phase I (TOHP-I)—weight reduction was the most effective strategy, producing a net weight loss of 3.9 kg and a BP change of −2.3/−2.9 mmHg [83]. TOHP-II [84] demonstrated that in overweight adults with high-normal BP, weight loss, and reduction in sodium intake, individually and in combination, were effective in lowering systolic and diastolic BP, especially in the short term (6 months). Furthermore, it concluded that the effects on average BP declined over time (48 months) and reductions in HTN incidence were achieved. The PREMIER clinical trial [85] reported that individuals with above-optimal BP, including stage 1 HTN, can make multiple lifestyle changes that lower BP and reduce their CVD risk. 

While all efforts must be taken to prevent DM/HTN, what about the people who already have these conditions? Unless proper care is provided to these individuals, they could face a huge burden of complications in the future. Three landmark studies on glycemic control in DM, namely, the Diabetes Complications and Control Trial (DCCT), the United Kingdom Prospective Diabetes Study (UKPDS), and Kumamoto Study [86–88], have clearly documented the beneficial effects of glycemic control in preventing microvascular complications in diabetic patients. However, mere glycemia control alone may not be sufficient to prevent CVD as shown in the UKPDS Study where despite a 16% reduction in MI, it did not reach statistical significance. Thus it is clear that a much prolonged approach controlling glucose, blood pressure, and lipids is needed to prevent CVD in diabetic subjects as shown by STENO-2 Study [89]. Indeed a tight DM control may even be harmful in older high risk patients as shown by the Action to Control Cardiovascular Risk in Diabetes (ACCORD) Trial [90], which showed that aiming for a glycated hemoglobin around 6.0% could result in increased mortality. Thus the overall consensus is that a glycated hemoglobin of less than 7% would be adequate [91]. Skyler et al. [92] have suggested that glycemic control may be important before macrovascular disease is well developed but has less impact when vascular disease is advanced. The same group recommends that for primary and secondary CVD risk reduction in diabetic patients, the evidence-based recommendations for blood pressure treatment, including lipid-lowering with statins, aspirin prophylaxis, smoking cessation, and healthy lifestyle behaviors outlined by American Heart Association (AHA) and the American Diabetes Association (ADA) guidelines [91, 93] should be followed.

 It is thus clear that it is important to improve levels of care at several health care settings, namely, primary, secondary, and tertiary. Unfortunately, there is an acute shortage of general physicians in rural areas. Thus at the primary level, the existing primary health centres (PHCs) and rural clinics must be strengthened. This includes providing basic training to the doctors and paramedical staff as well as community health workers as well as improving the facilities available at the PHCs, for example, essential medicines, glucometers, blood pressure apparatus, and so forth. Screening can be successfully done by nonphysicians as shown in the Chunampet Rural Diabetes Prevention Project (CRDPP) [94]. Large scale screening for DM/HTN is possible, but it then becomes an ethical issue to provide basic care, for example, low cost generic drugs and follow-up care. 

Studies in which the effectiveness of professional interventions (postgraduate education of health care) combined with local consensus procedures and/or reminders and/or audit and feedback were compared with usual care have reported an improved diabetes care [95, 96]. Another study which evaluated education for both health care professionals and patients showed that the intervention improved glycated hemoglobin, body weight, and triglycerides [97]. Proper referral systems for treatment must also be available. Measures should be taken to increase the adherence to practice guidelines and improve both primary care doctors' and patients' knowledge. Evaluating and auditing of adherence to the national guidelines and management in primary healthcare should be done regularly to ensure that appropriate care is provided. There is ample evidence from many developed and developing countries to show that type 2 DM and HTN are routinely managed by nurses/health workers/pharmacists in primary care centres in conjunction with a physician through patient education, patient and family counseling, and close monitoring of health outcomes [98–103]. Mexico's Veracruz Initiative for DM awareness (VIDA) showed the potential of implementing chronic disease management principles in low-resource primary healthcare settings, where health workers received in-service training on DM management, including foot care, and also learned about principles of effective health care for chronic disease as represented by the chronic care model [102]. A similar approach has been adopted in rural South Africa for DM, HTN, and asthma and resulted in successful control of the disorders [103]. 

Application of technology can also be used to control the twin epidemic. Telemedicine, which is an integration of electronic information and medical technology, could help to bring specialized healthcare to the remotest and underserved areas in developing countries [104]. Telemedicine is an exciting new technology which can help in integration of all urban and rural health care centers and improve the quality of medical services in the presently underserved and impoverished sections particularly in remote rural areas of developing countries. The potential of this tool is particularly significant in SEA and AFR regions where specialists are few and where distances and the quality of the infrastructure hinder the movement of physicians or patients. There is evidence in both of these regions to show that this technology can nullify the gap in providing quality health care where health care professionals cannot reach out to people with chronic disease who need their services [94, 105–108]. The Chunampet Rural Diabetes Prevention Project (CRDPP), which was developed to provide mass screening and DM health care and prevention to rural India through a combination of telemedicine and personalized care and employment of local youth (both men and women), demonstrated that the model is not only suitable for prevention and control of DM but also reduces the burden of other chronic NCDs such as HTN in low-resource rural settings [94]. Thus, novel methods such as this must be developed to provide health care, in general, and, in particular, DM and HTN prevention, which is accessible, affordable, and acceptable to rural people in developing countries.

 In addition, the existing infrastructure at government hospitals, which provide secondary level of care, should be improved to screen and provide basic treatment for DM-related complications or CVD/stroke in addition to some laboratory support, for example, lipid profile, glycated hemoglobin, microalbuminuria, or serum creatinine estimation and ECG. In addition, Public Private Partnerships could be encouraged if the Government cannot provide all the necessary infrastructure or personnel to accomplish this [109]. Providing large scale insurance benefits to those below the poverty line can help to prevent them from getting into a “debt trap” because of illness-related health care expenditure [110]. 

A small percentage of individuals (about 5%) will ultimately need tertiary care facilities for management of their advanced stage of complications [94], for example, Laser photocoagulation for diabetic retinopathy, dialysis or transplantation for renal failure, coronary angiography or bypass/angioplasty for CAD, or limb salvage procedures or amputations for advanced diabetic foot disease (tertiary prevention). For these procedures, help of tertiary care centres would be needed. Again, costs, quality control, and accountability could be ensured by mutually acceptable terms and some or all of these costs could be defrayed by insurance schemes specifically meant to benefit people.  Table 4 summarizes some of the action plans to prevent/control DM and HTN.

According to the United Nations Joint Program on HIV/AIDS, 33.2 million adults and children are living with these infections worldwide. Of these, two to three million are estimated to be in South Asia [111]. Many low- and middle-income countries, particularly the AFR region, face a double burden of disease from infectious diseases such as HIV/AIDS and NCDs [112]. The health systems in such countries are weak and are severely challenged by the weight of a double burden of disease. Recently, a compelling case has also been made for coordinated or “diagonal” approach to HIV/AIDS and NCD prevention and control as they share many similarities that make them ideal candidates for a coordinated approach [112–114]. There is paucity of data on the integration of care for HIV, DM, and CVD in several low-income and middle-income countries although they coexist [115]. A review on care delivery models for DM and HIV/AIDS in Sub-Saharan Africa revealed potential elements for cross-fertilization which includes rapid scale-up approaches through the public health approach by simplification and decentralization; community involvement, peer support, and self-management strategies; and strengthening health services [112]. A Cambodian study demonstrated the feasibility of integrating care for HIV/AIDS, DM, and HTN within the setting of chronic disease clinics and reported over 70% retention of patients after 24 months [116]. HIV/AIDS models have a lot of experience with activating community and peer support for patients, which can be followed in prevention of chronic diseases including HTN and DM. Therefore, lessons from these studies reveal that there is no need to reinvent the wheel, as experiences with HIV programmes can be leveraged to NCD programmes, and vice versa. With appropriate resources and support, such approaches could be used in primary health care.

## 6. Conclusion

**Figure 3:**
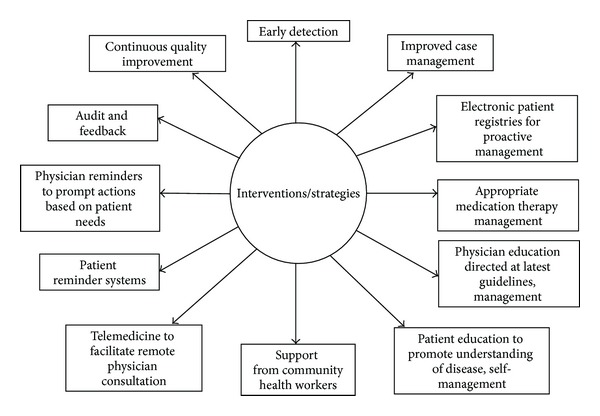
Interventions/strategies to improve health care for chronic disease.

Both DM and HTN present huge challenges in developing countries, particularly in the SEA and AFR regions. Health care systems should be strengthened for early detection and effective treatment of those affected with both DM and HTN. Strategies to prevent/control HTN and DM should be population based, incorporating multilevel, multicomponent, and socioenvironmental approaches and integrating community resources with public health and clinical care. According to the chronic care model put forth by WHO in 2002, six areas need improvement to facilitate chronic care: community resources; health systems; self-management support; decision support; delivery system redesign; and clinical information systems [117], all of which give greater emphasis to the need for policy changes and empowering the community [118]. Based on this, various interventions/strategies have been suggested to improve health care for chronic disease as shown in Figure 3. There is an urgent need to improve monitoring and management of risk factors through primary care linked programmes. Policy and system changes are essential to reduce risk in populations, including legislation and public education to reduce dietary fat, salt and sugar, food pricing policies, tobacco control, and changes to health care delivery systems to explicitly support prevention/control of DM and HTN. Ultimately, prevention of DM/HTN needs “political will”, societal and community support, and of course behavioral change on the part of the individuals and their families.

## Figures and Tables

**Table 1:** Number of people with diabetes (in thousands) in the 20–79 age group in countries of Southeast Asia (2011 and 2030) [3].

Region	2011	2030
Southeast Asia (SEA)	**71400**	**120900**
Bangladesh	8406	16837
Bhutan	22	41
DPR Korea	1508	1934
India	61258	101203
Indonesia	7292	11802
Maldives	15	32
Myanmar	2104	3482
Nepal	488	1171
Sri Lanka	1080	1467
Thailand	4014	5454
Timor-Leste	30	55

**Table 2:** Number of people with diabetes (in thousands) in the 20–79 age group in countries of African region (2011 and 2030) [3].

Country	2011	2030
Algeria	1435	2351
Angola	185	383
Benin	71	143
Botswana	94	134
Burkina Faso	175	371
Burundi	94	191
Cameroon	501	913
Cape Verde	13	25
Central African Republic	58	99
Chad	197	401
Comoros	23	48
Congo	95	176
Cote d'Ivoire	407	813
DR Congo	731	1422
Equatorial Guinea	14	26
Eritrea	95	192
Ethiopia	1377	2629
Gabon	69	124
Gambia	12	26
Ghana	517	1036
Guinea	182	324
Guinea-Bissau	19	33
Kenya	769	1683
Lesotho	29	52
Liberia	51	97
Madagascar	428	832
Malawi	352	747
Mali	100	217
Mauritania	61	119
Mauritius	138	196
Mozambique	295	581
Namibia	74	135
Niger	284	620
Nigeria	3055	6113
Rwanda	126	275
Sao Tome and Principe	4	8
Senegal	146	296
Seychelles	4	5
Sierra Leone	72	127
South Africa	1947	2548
Swaziland	14	22
Togo	81	153
Uganda	308	690
United Republic of Tanzania	473	1107
Zambia	244	432
Zimbabwe	551	1053

**Table 3:** Trends in age-standardized mean SBP in selected Africa region between 1980 and 2008 for male and female population [6].

Selected Africa region	Mean systolic blood pressure (mmHg)							
	Female				Male			
Year	1980	1990	2000	2008	1980	1990	2000	2008
Algeria	130.8	131	130.5	129.9	132.4	130.4	129.2	130
Angola	130.1	129.9	129.9	130.1	135.2	133.6	132.4	133.6
Cameroon	125.9	125.9	125.6	127.3	132.8	130.1	128.8	131.3
Cote d'Ivoire	130.3	130.5	130.7	131.5	136.5	133.2	132.7	134.6
DR Congo	129.7	129.3	128.1	129.4	134.0	131.6	130.4	132.7
Ethiopia	116.6	120.1	123.3	126.6	123.1	124.5	126.4	129.6
Ghana	125.7	127.6	129	128.3	130.5	129	129.1	129.5
Kenya	123.7	124.9	127.3	129.9	128.2	127.7	128.9	132.1
Mozambique	127.8	130.0	132.6	135.4	133.4	132.5	134	137.5
Nigeria	131	131.8	133.9	135.5	134.5	130.1	130	132.8
South Africa	133.2	132.3	130	131	135.4	133.6	131.3	133.8
Uganda	126.3	129.2	132.0	134.5	130.7	130.7	132.4	135.6
United Republic of Tanzania	122.6	125.6	127.9	130.8	126.4	126.4	128.2	131.6
Zimbabwe	128.7	130.4	130.8	132	130.8	130.3	130.2	131.9

**Table 4:** Action plans for prevention/control of diabetes and hypertension in different health care settings.

	Detection	Diabetes education	Equipment	Medicines
Primary care	Large scale screening using high risk category approach	Through community workers—training of community workers in screening activities	Basic equipment like Glucometer, BP apparatus	Essential low cost generic drugs to be made available at all PHCs

Secondary care	Confirmation of diagnosis Screening for complications of diabetes and co-morbid conditions	(i) Physicians (ii) Paramedicals	Ophthalmology Basic laboratory support	Insulin, oral hypoglycemic drugs, ACE inhibitors, calcium antagonists, ARBs, statins, aspirin, and other antihypertensive drugs

Tertiary care	Treatment of complications	(i) Diabetologists (ii) Ophthalmologists (iii) Nephrologists (iv) Cardiologists	Advanced equipment (i) Laser photocoagulation (ii) Fundus camera (iii) Cardiac care (iv) Dialysis unit (v) Transplantation	All of above and costlier drugs or treatments
